# Role of miRNAs and lncRNAs in hematopoietic stem cell differentiation

**DOI:** 10.1016/j.ncrna.2020.12.002

**Published:** 2020-12-19

**Authors:** Soudeh Ghafouri-Fard, Vahid Niazi, Mohammad Taheri

**Affiliations:** a Urogenital Stem Cell Research Center, Shahid Beheshti University of Medical Sciences, Tehran, Iran; b Department of Tissue Engineering and Applied Cell Sciences, School of Advanced Technologies in Medicine, Shahid Beheshti University of Medical Sciences, Tehran, Iran; c Urology and Nephrology Research Center, Shahid Beheshti University of Medical Sciences, Tehran, Iran

**Keywords:** miRNA, lncRNA, Hematopoietic stem cells

## Abstract

Non-coding RNAs (ncRNAs) have diverse roles in the differentiation of hematopoietic cells. Among these transcripts, long ncRNAs (lncRNAs) and microRNAs (miRNAs) have especial contribution in this regard particularly by affecting levels of transcription factors that define differentiation of each linage. miR-222, miR-10a, miR-126, miR-106, miR-10b, miR-17, miR-20, miR-146, miR-155, miR-223, miR-221, miR-92, miR-150, miR-126 and miR-142 are among miRNAs that partake in the differentiation of hematopoietic stem cells. Meanwhile, this process is controlled by a number of lncRNAs such as PU.1-AS, AlncRNA-EC7, EGO, HOTAIRM1, Fas-AS1, LincRNA-EPS and lncRNA-CSR. Manipulation of expression of these transcripts has functional significance in the treatment of cancers and in cell therapy. In this paper, we have provided a brief summary of the role of miRNAs and lncRNAs in the regulation of hematopoietic stem cells.

## Highlights

• Non-coding RNAs have diverse roles in the differentiation of hematopoietic cells.
• Manipulation of expression of these transcripts has functional significance in the treatment of cancers and in cell therapy.

## 1 Introduction

**Fig. 1:**
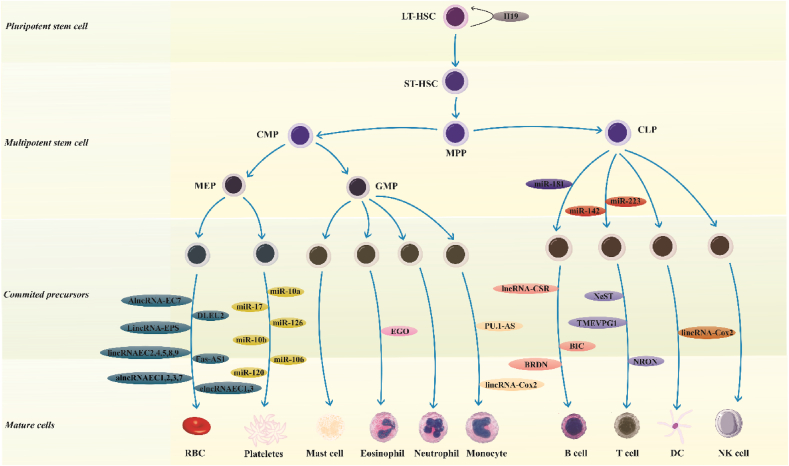
Non-coding RNAs (ncRNA) include abundant small regulatory RNAs namely microRNAs (miRNAs), in addition to lots of polyadenylated and non-polyadenylated long ncRNAs (lncRNAs). Currently, ncRNAs are proven as regulators of hematopoiesis and leukemogenesis [6]. Figure represents the most important ncRNA contributing in the differentiation of hematopoietic stem cells into functional blood cells [7,8].

Non-coding RNAs (ncRNAs) have diverse roles in the biologic processes. Compared with the mRNA-coding transcripts, ncRNA transcripts more abundant in the human genome [1]. Two groups of ncRNAs have attracted attention of researchers due to their regulatory roles on the expression of genes. These groups of transcripts are long ncRNAs (lncRNAs) and microRNAs (miRNAs) [1]. In addition to acting as enhancers of transcription, lncRNAs can function as signals, decoys, scaffold transcripts and guide transcripts to directly regulate gene expression or recruit other regulatory molecules to alter gene expression [2]. The regulatory role of miRNAs on gene expression is exerted via their incorporation into the RNA-induced silencing complex (RISC). Subsequently, they can decrease expression of their targets. Based on the extent of similarity between the miRNA and target sequences, they degrade mRNA or inhibit its translation [3]. Both lncRNAs and miRNAs can regulate differentiation of hematopoietic cells [4,5]. Fig. 1 represents a summary of ncRNAs with critical roles in the differentiation of hematopoietic cells.

In the present review, we have provided a brief record of the role of miRNAs and lncRNAs in the regulation of HSCs.

## 2 miRNAs role in differentiation of HSCs

After assessment of miRNA signature in normal human bone marrow, Georgantas et al. have described expression of more than 30 miRNAs in CD34^+^ hematopoietic stem-progenitor cells (HSPCs). Subsequently, they integrated miRNA signature with mRNA profile of these cells and predicted miRNA-mRNA interaction data. Among the identified miRNAs has been miR-155, a miRNA that can regulate myelopoiesis and erythropoiesis. miRNA-155 has been shown to decrease both myeloid and erythroid colony construction from HSPCs [9]. Another pioneer study in this field has shown the role of various miRNA families in controlling HSC self-renewal and differentiation with HSCs being described by a certain miRNA profile in each differentiation phase. For instance, expressions of miR-125a, miR-125b, miR-155, miR-99a, miR-126, miR-196b, miR-130a, miR-542–5p, miR-181c, miR-193b and let7e have been significantly increased in long term-HSCs (LT-HSCS) [10]. Over-expression of miR-125b-5p, miR-126–3p and miR-155 in bone marrow cells has led to a competitive engraft enhancement in the bone marrow in all downstream lineages, while miR-196b, miR-181c, let7e and miR-542–5p have conferred an opposite effect. These observations have suggested the functional effect of these miRNAs in the regulation of HSC homeostasis instead of a certain role in differentiation to some specific phenotypes [10].

Using a high throughput combinatorial technique, Petriv et al. have assessed miRNA signature in 27 different cell populations and categorized these cells based on miRNA profile into six chief groups namely stem cell populations and multipotent progenitor cells, lymphoid cells, and four diverse principal classes of myeloid cells. They have reported alterations in the expressions of numerous miRNAs at distinctive nodes. Notably, miR-125b, miR-196a/b, miR-130a, let-7d, miR-148b and miR-351 have been the utmost differentially expressed miRNAs between stem cell populations and progenitor cells compared with the more mature cells [11].

Chen et al. have reported specific expression of three miRNAs in the hematopoietic cells. They have also demonstrated dynamic regulation of their expression throughout early hematopoiesis and lineage definition. Among these small transcripts, miR-181 has been mostly expressed in the B-lymphoid cells, and its expression in HSPCs has resulted in the preferential expansion of B-lineage cells [12]. miR-23a cluster is also involved in the regulation of lymphopoiesis since deficiency of this cluster in mice has resulted in the enhancement of B lymphopoiesis at the cost of myelopoiesis. However, HSPCs have not been altered. Concomitant deletion of mirn23a and mirn23b in adult bone marrow has also twisted HSPC differentiation to B cells at the cost of myeloid cells. Notably, double-knockout of these miRNAs has reduced bone marrow cellularity and diminished HSC and HSPC populations, demonstrating the exacerbation of the phenotype detected in mirn23a deficient mice [13]. On the other hand, miR-29a has a prominent role in controlling differentiation of myeloid lineage. This miRNA is over-expressed in early progenitors contributing in preservation of the undifferentiated status, whereas its expression has been decreased in the course of differentiation [14]. Therefore, forced over-expression of miR-29a in mice HSCs has conferred self-renewal aptitudes of myeloid precursors, enhancing myelopoiesis [14]. Another study has demonstrated the impact of miR-125b over-expression in bone marrow in induction of a myeloproliferative condition that might lead to myeloid leukemia [10].

Felli et al. have shown the role of miR-221 and miR-222 in reduction of proliferation of CD34^+^ progenitors and enhancement of differentiation of erythropoietc cells. These effects have been complemented by a significant reduction of kit protein. Besides, miR-221 and miR-222 treated CD34^+^ cells had lower engraftment capability and impaired stem cell activity upon transplantation in NOD-SCID animals. Taken together, under-expression of miR-221 and miR-222 increases kit protein synthesis, therefore resulting in the development of early erythroblastic cells [15]. Garzon et al. A high throughput expression profiling of CD34+-derived megakaryocytes has shown under-expression of miR-10a, miR-126, miR-106, miR-10b, miR-17 and miR-20. miR-130a has been shown to alter expression of MAFB, a transcription factor which stimulates expression of platelet-related protein GPIIB. Besides, miR-10a reduces expression of HOXA1. Evaluation of miRNA signature in the megakaryoblastic leukemic cells and in vitro differentiated megakaryocytes has demonstrated over-expression of miR-101, miR-126, miR-99a, miR-135, and miR-20 in the former cells [16]. Fazi et al. have uncovered the role of miR-223, NFI-A and C/EBPα in the regulation of differentiation of human granulocytes. They have also demonstrated a competition between NFI-A and C/EBPα for binding with promoter of miR-223. While NFI-A retains miR-223 expression low, C/EBPα enhances miR-223 expression after induction of cell differentiation by retinoic acid. Therefore, miR-223 participates in the process of granulopoiesis. It also down-regulates NFI-A expression to further mediate gene reprogramming in the granulocyte lineage [17]. miR-150 is among miRNAs with specific expression in the hematopoietic cells. This miRNA has been shown to be predominantly expressed in the lymph nodes and spleen, being over-expressed in the course of development of mature T and B cells with a sharp up-regulation in the immature B cell phase. Forced up-regulation of miR-150 in HPSCs has impaired the development of mature B cells, with no significant effects on the development of mature CD8^+^ and CD4^+^ T cells, granulocytes or macrophages upon transplantation. Besides, early expression of miR-150 has obstructed the conversion of pro-B cells to the pre-B cell lineage. Taken together, miR-150 possibly inhibits expression of transcripts that have critical roles in development of pre- and pro-B cells [18]. miR-150 has been predicted to target c-Myb, a transcription factor governing numerous stages of lymphocyte expansion. miR-150 precisely regulates c-Myb expression to fundamentally influence lymphocyte development [19]. miR-125a is another miRNA whose role in enhancing the number of HSCs has been displayed *in vivo*. This process is completed via a specific inhibition of apoptosis in immature progenitors of this lineage, probably through regulation of expression of a number of pro-apoptotic gene targets among them is Bak1 which is directly targeted by miR-125a [20]. Table 1 sums up the results of investigations that appraised the role of miRNAs in HSC differentiation.

**Table 1:** Role of miRNAs in hematopoietic stem cell differentiation.

miRNA	Cell linage	Function	Reference
miR-181	HSCs/HPCs & Pro-B lymphocyte	Attach to CXCR4 and Induces B-lymphocyte differentiation	[9,12]
miR-222	HSCs/HPCs	Attach to FOS, cKIT and Blocks erythropoiesis	[9,15]
miR-10a, 126, 106, 10b, 17, 20	megakaryocyte	Regulate megakaryocyte differentiation	[16]
miR-146	T helper lymphocyte	Block differentiation of T helper lymphocyte	[9]
miR-155	HSCs/HPCs	Attaches to CREBBP, MEIs1, PU.1,AGTR2 and FOS and blocks differentiation	[9]
miR-223	HSCs/HPCs & Pro T cell	Attach to NFI-A and increase granulopoiesis and induces T lymphocyte lineage	[9,12,17,21]
miR-221	HSCs/HPCs	Attaches to FOS and cKIT and blocks erythropoiesis	[9,15]
miR-92	HSCs/HPCs	Attach to KLF	[9]
miR-150	B cell and T lymphocyte	Downregulates C-MYB and control proliferation and differentiation of B cell and T lymphocyte	[18,19]
miR-126	HSCs/HPCs	Decrease self-renewal and enhance mobilization of HSCs	[22,23]
miR-142	ProT lymphocyte	Induces T lymphocyte lineage	[12]
miR-125a	HSCs/HPCs	Was increased in HSC and decreases apoptosis by targeting the Bak1.	[24]
miR-29a	HSCs/HPCs	Affects common myeloid progenitors and granulocyte macrophage progenitors; Induces myeloid biased differentiation	[25]
miR-133	MSC	Blocks MSC differentiation	[26]
miR-196b	HSCs/HPCs	Has a negative effect on the engraftment of bone marrow	[27]
miR-29a	HSCs/HPCs	Downregulates actin-binding protein; regulate early HSCs; Was highly expressed in HSCs/HPCs	[25,28]
miR-130	HSCs/HPCs	Was enriched in long term HSC; increases self-renewal	[27]
miR-34a	Pro B lymphocyte	Inhibition of Foxp1; regulation of pro-B to pre-B by miR-34a	[29]
miR-299–5p	Megakaryocyte	Modulates megakaryocyte differentiation	[30]
miR-23a/b	HSCs/HPCs	Proper proliferation and differentiation of HSCs/HPCs	[31]
miR-15/16	HSCs/HPCs	Erythroid differentiation	[32]
miR-21	HSCs/HPCs	Myelopoiesis	[33]
miR-22	HSCs/HPCs	HSC maintenance	[34]
miR-145/miR-146a	HSCs/HPCs	Involved in megakaryopoiesis	[35]
miR-28	HSCs/HPCs	Prevents megakaryocyte differentiation	[36]
miR-27a	megakaryocyte	Attaches to RUNX1 and decreases its levels	[37]
miR-144, miR-451	HSCs/HPCs	Erythroid homeostasis	[38,39]
miR-451	HSCs/HPCs	Erythroid differentiation	[40]

## 3 LncRNAs role in differentiation of HSCs

The function of lncRNAs in the differentiation of HSPCs has been investigated in numerous studies. LncRNAs can regulate expression of transcription factors which regulate hematopoiesis. Luo et al. have assessed lncRNA profile of HSCs by high throughput sequencing and recognized more than 300 unannotated lncRNAs. Comparison of expression of these lncRNAs in differentiated lineages has led to identification of 159 HSC-enriched lncRNAs (LncHSCs). Silencing of two LncHSCs has conferred specific impact on HSC self-renewal and lineage commitment possibly through modulation of principal hematopoietic transcription factor, namely E2A [41]. Expression of the transcription factor PU.1 has been controlled by an antisense lncRNA which is transcribed from the same locus namely PU.1-AS. This lncRNA has been shown to suppress *PU.1* expression through regulating its translation [42]. Notably, others have described that high level of PU.1 is required for the development of macrophage compared with neutrophils [43]. Therefore, fine-tuning of PU.1 expression by its antisense transcript might define the lineage development. Paralkar et al. have identified more than 1000 polyadenylated lncRNAs expressed in erythroblastic cells, megakaryocytes, and megakaryocyte-erythroid precursor cells of mouse, and about 600 lncRNAs in human erythroblasts. The majority of these lncRNAs have been shown to be controlled by chief transcription factors including GATA1 and TAL1 [44]. Wagner et al. have reported over-expression of EGO in human bone marrow and in mature eosinophilic cells. This lncRNA has been shown to be transcribed from an intronic region of the *ITPR1* gene. Stimulation of CD34^+^ hematopoietic progenitors with IL-5 has enhanced expression of EGO. EGO knock down has reduced expression of MBP and EDN in developing CD34^+^ hematopoietic progenitors [45]. HOTAIRM1 is another antisense transcript originating from the same CpG island that is around the initiation site of *HOXA1* gene. HOTAIRM1 is the most noticeable intergenic RNA which is over-expressed in the course of induced granulocytic differentiation of hematopoietic cells. This lncRNA contributes in the myelopoiesis cia regulation of HOXA cluster [46]. Expression of Fas-AS1 has also been induced in the course of erythropoiesis via the activity of important erythroid transcription factors GATA-1 and KLF1. This lncRNA is inhibited by NF-κB. Besides, up-regulation of Fas-AS1 in HSPCs-originated erythroblasts has decreased surface levels of Fas and induced defense against Fas-mediated apoptosis [47]. LincRNA-EPS has a role in the erythroid differentiation as its suppression has blocked erythroid differentiation and enhanced apoptosis. This lncRNA has been shown to suppress expression of the pro-apoptotic gene *Pycard* [48]. Linc-MAF-4 is a chromatin-related lncRNA with specific expression in T helper 1 cells. Its expression has been inversely correlated with expression of the T helper 2-associated transcription factor MAF. Linc-MAF-4 silencing has twisted T cell differentiation to the T helper 2 route [49]. H19 is another lncRNA with critical role in the emergence of HSCs. Absence of H19 in the early developmental stages has suppressed endothelial-to-hematopoietic transition. Besides, H19 deficiency in pre-HSCs has resulted in promoter hypermethylation and simultaneous down-regulation of numerous important hematopoietic transcription factors, such as Runx1 and Spi1. The detected defects in the hematopoietic system following H19 deficiency has been attributed to the enhanced function of S-adenosylhomocysteine hydrolase, a controller of DNA methylation [50]. An animal study has indicated the role of Xist RNA in the suppression of hematologic cancer as deletion of this lcRNA in the blood of mice has resulted in initiation of an extremely aggressive myeloproliferative condition being described by a number of characteristics including myelofibrosis and leukemia. Deficiency of this lncRNA in HSCs has resulted in abnormal maturation and age-dependent defects [51]. Dlk1-Gtl2 is another ncRNA with an important impact in inhibition of LT-HSCs. This locus contains a miRNA mega-cluster locus that inhibits the whole PI3K-mTOR pathway, suppressing mitochondrial synthetic processes and metabolic function and protecting LT-HSCs from reactive oxygen species (ROS) [52]. Table 2 reviews the investigations that assessed the role of lncRNAs in HSC differentiation.

**Table 2:** Influence of lncRNAs in hematopoietic stem cell differentiation.

LncRNA	Full name	Cell linage	Function	Reference
PU.1-AS	*	Monocytes; macrophages	Regulates translation of PU.1 in HSCs differentiation	[42,43]
AlncRNA-EC7	*	erythrocyte	Downregulates expression of BAND3 and inhibit maturation of erythrocyte	[44]
AlncRNA-EC3	*	erythrocyte	Modulate red blood cell (RBC) formation	[53]
ShlncRNA-EC6	*	erythrocyte	Promotes red blood cell maturation	[53]
EGO	Eosinophil granule ontogeny	Leukocyte maturation	Modulates MBP in the development of HSCs CD34^+^	[45]
HOTAIRM1	HOX antisense intergenic RNA myeloid 1	Myeloid progenitors	Modulation of granulocytic differentiation genes and the neighboring 3′ HOXA genes in HSCs	[46,54,55]
HOTAIRM1	HOX antisense intergenic RNA myeloid 1	Leukocyte	Absence of HOTAIRM1causes ATRA-induced myeloid differentiation.	[56,57]
Fas-AS1 (or Saf)	Fas-antisense 1	erythrocyte	During erythropoiesis some erythroid transcription factors such as GATA-1 and KLF1 overexpress Fas-AS1	[47,58]
LincRNA-EPS	LincRNA erythroid prosurvival	erythrocyte	Downregulates expression of PyCARD and enhance erythropoiesis	[48,53]
Rmrp	*	Th17 CD4^+^ T	Change the expression of RORgt transcription factor in the Th17	[59,60]
lncRNA-CSR	LncRNA-class switch DNA recombination	B lymphocyte	Regulates function of lymphocyte B and antibody secretion	[61]
NeST (Tmevpg1 or IFNG-AS1)	Nettoie Salmonella pas Theiler's;	Th1 CD4^+^ T	In Th1 lymphocyte, NeST Binds to WDR5 and changes histone 3 methylation.	[62,63]
Linc-MAF-4	*	Th1 CD4^+^ T	Changes T- lymphocyte differentiation toward Th2 by the change in MAF transcription that alters the function of chromatin modifiers	[49]
LincR-Ccr2-5′AS	*	Th2 CD4^+^ T	Changes the expression of specific genes that modulate the migration of Th2	[64,65]
GATA3-AS1	GATA3-Antisense1	Th2 CD4^+^ T	Regulation of Th2- lymphocyte	[66]
TH2-LCR	TH2-locus control region	Th2 CD4^+^ T	Regulates the secretion of cytokines in Th2- lymphocyte	[67]
LncRNA-CD244	*	CD8^+^ T	Changes expression of IFN-g and TNF-a and modify function of lymT CD8^+^	[68,69]
NRON	noncoding (RNA) repressor of NFAT	T lymphocyte	Regulation of NFAT1 transcription factor	[70]
BIC	B- lymphocyte integration cluster	B lymphocyte	Regulator of B- lymphocyte differentiation	[[71], [72], [73], [74]]
Flicr	Foxp3 long intergenic non-coding RNA	Treg	Modulates Treg functions, strength antiviral responses	[75]
Lnc-EGFR	Lnc-epidermal growth factor receptor;	Treg	Changes the differentiation of Treg and induced immunosuppression	[76]
lincRNA-Cox2	*	Dendritic cells; macrophages	Regulate secretion of IFNs	[77]
CRNDE	Colorectal neoplasia differentially expressed	B lymphocyte	Regulates function of primarily pre-B1, pre-B2, and centroblasts	[78]
NeST	*	T lymphocyte	Regulates immune function of T lymphocyte	[79,80]
LincR-Ccr2-5′ AS	*	T lymphocyte	Regulation of Ccr1, Ccr2, Ccr3, and Ccr5 genes	[81]
Thy-ncR1	*	Thymic T lymphocyte	Destruction of MFAP4 and modulate proliferation and differentiation of T-cell	[82]
TMEVPG1	*	T lymphocyte	Changes the expression of IFN-γ gene and modify proliferation and differentiation of T- lymphocyte	[77,80]
H19	*	HSC	Preserves long-term HSC quiescence and self-renewal	[83]
EGO	Eosinophil granule ontogeny	Eosinophils	Regulates eosinophils differentiation genes and maturation of eosinophils	[84]
HOTAIRM1	*	Myeloid progenitors	Suppuration of HoxA1 and HoxA4 genes in myeloid progenitors	[45]
LincRNA-EPS	*	Erythroblasts	Elevates apoptosis	[46]
DLEU2; elncRNAEC1,3; lincRNAEC2,4,5,8,9; alncRNAEC1,2,3,7	*	Erythroblasts	Regulates erythrocyte maturation	[48]
Dlk1-Gtl2 Locus-derived lncRNAs	*	HSC	lncRNAs inhibit PI3K-mTOR signaling, resulted in maintain HSC self-renewal	[52]
lncRNA Evx1	*	Pluripotent cells	Binds to chromatin and increases EVX1 transcription; regulate gene expression, proliferation, and differentiation	[85]
lncRNA-H19	*	Embryonic HSC	Partakes in endothelial-to-HSC transition by regulation of transcription factors (Runx1 and Spi1)	[86]
lncHSC-1/2	Hematopoietic stem cell	HSC	Controls long-term HSC quiescence and self-renewal	[6]
lncRNA-Xist	*	HSC	Regulates HSC quiescence and self-renewal	[51,87]
lncRNA-DC	Dendritic cells	DC	Regulates DC differentiation by increasing phosphorylation and nuclear translocation of STAT3	[88]
lncRNA- Lethe	*	Macrophage/DC	Partakes in innate immune response; regulate and limit inflammation	[89]
lincRNA-Cox2	*	Macrophage/DC	Is induced downstream of Toll-like receptors (TLRs) activation; act in the innate immune response	[81,89]
lncRNA-THRIL	TNF- and hnRNPL-related immunoregulatory lncRNA	Macrophage/DC	Regulates homeostasis and activation of inflammatory reaction; necessary for expression of inflammatory cytokines	[90]
lncRNA-PACER	p50-associated COX-2 extragenic RNA	Macrophage/DC	Has an important role in decoy molecule in the NF-kB signaling pathway	[91]
lncRNA-NKILA	NF-kB-interacting lncRNA	Macrophage/DC	Regulates NF-kB signaling pathway; induced after IL-1b and TNF-a stimulation	[92,93]
lncRNA-αGT	α-globin transcript	erythrocyte	Differentiation of erythroid cells	[94]
lncRNA- GAS5	*	HSC	Act as a tumor suppressor lymphoma and leukemia	[95]
lincRNA-a7	*	HSC	Regulation of hematopoiesis	[96]
lncRNA-MEG3	*	HSC	Regulation of p53 gene	[97]
lncRNA-NRON	*	HSC	Regulating the activity of NFATs	[98]
lncRNA-Morrbid	*	Myeloid cell	Controls myeloid cell differentiation	[48]
lnc-MC	*	Monocyte/Macrophage	Regulates monocyte/macrophage differentiation	[99]

Th: lymphocyte T helper; Treg: lymphocyte T regulatory; NFAT1; nuclear factor of activated T-cells 1, MFAP4; microfibril associated protein 4, IFNs; interferon, STAT3: signal transducer and activator of transcription 3, DC: Dendritic cells, NFATs: nuclear factor of activated T cells.

## 4 Discussion

NcRNAs have critical regulatory functions in cell proliferation, programmed cell death, organ development, and differentiation. Both miRNAs and lncRNAs are important elements of the molecular pathways that regulate hematopoiesis. A number of these transcripts influence the expression of transcription factors that regulate differentiation of certain lines of hematopoietic cells. Few antisense transcripts have been identified that modulate expression of transcription factors *in cis*. Identification of other overlapping complementary transcripts with regulatory roles on the expression of transcription factors would facilitate clarification of molecular mechanisms of HSPCs differentiation. The majority of lncRNAs in the hematopoietic cells which have been identified through high throughput methods are unannotated, highlighting the prospect for novel discovery via investigating specialized cell kinds [44]. Several of lncRNAs which are extensively expressed during erythropoiesis have been shown to be controlled by critical erythroid transcription factors such as GATA1, TAL1, or KLF1 [53], revealing the mutual interactions between transcription factors and lncRNAs.

Notably, a vast body of literature about the contribution of ncRNAs in the differentiation of hematopoietic cells has come from the animal studies. Although these studies have provided invaluable clues about this subject, verification of their results in the human cells is a necessary step for implementations of these results in the clinical settings. Few comparative studies have demonstrated lack of conservation of hematopoietic cell-associated lncRNAs between mammalian species [44], signifying the importance of assessment of expression of these transcripts in each species.

Notably, exosomes originated from HSPCs have been shown to encompass ncRNAs, therefore transferring these transcripts to the recipient cells to modulate their function [23]. Exosome-mediated transfer of ncRNAs represents an important way of modulation of bone marrow microenvironment.

High throughput sequencing methods have shown significant differences in the miRNA profile between hematopoietic and non-hematopoietic cells. In addition, miRNA signature is slightly different within the hematopoietic group. Notably, completely differentiated effector cells and precursors at parallel stages of differentiation share miRNA pattern to a high extent. Therefore, miRNAs have critical functions during hematopoietic cell differentiation and in the process of maintenance of characteristics of different cells [100]. Some miRNAs have been shown to be specifically expressed in mature hematopoietic cells, but not their progenitors [19], thus regulating certain stages of development of hematopoietic cells. It is possible that miRNAs regulate the expression of only limited numbers of crucial target proteins in specific cellular settings [19]. Besides, miRNAs have a cell-stage-specific regulatory role in HSCs through which they control the stem cell bulk [20].

Manipulation of expression of these transcripts has functional significance in the treatment of cancers and in cell therapy. *In vitro* studies have shown the effects of silencing or over-expression of a number of ncRNAs in changing the differentiation process of hematopoietic cells, suggesting these methods as putative enrichment strategies before bone marrow transplantation.

## Declaration of competing interest

The authors declare they have no conflict of interest.

## References

[1] Diamantopoulos M.A., Tsiakanikas P., Scorilas A. (2018). Non-coding RNAs: the riddle of the transcriptome and their perspectives in cancer. Ann. Transl. Med..

[2] Fang Y., Fullwood M.J. (2016). Roles, functions, and mechanisms of long non-coding RNAs in cancer. Dev. Reprod. Biol..

[3] Macfarlane L.-A., Murphy P.R. (2010). MicroRNA: biogenesis, function and role in cancer. Curr. Genom..

[4] Li W., Ren Y., Si Y., Wang F., Yu J. (2018). Long non-coding RNAs in hematopoietic regulation. Cell Regen..

[5] Bissels U., Bosio A., Wagner W. (2012). MicroRNAs are shaping the hematopoietic landscape. Haematologica.

[6] Luo M., Jeong M., Sun D., Park H.J., Rodriguez B.A., Xia Z. (2015). Long non-coding RNAs control hematopoietic stem cell function. Cell stem cell.

[7] Tian X., Tian J., Tang X., Ma J., Wang S. (2016). Long non-coding RNAs in the regulation of myeloid cells. J. Hematol. Oncol..

[8] Mayani H. (2016). The regulation of hematopoietic stem cell populations. F1000Research.

[9] Georgantas R.W., Hildreth R., Morisot S., Alder J., Liu C-g, Heimfeld S. (2007). CD34+ hematopoietic stem-progenitor cell microRNA expression and function: a circuit diagram of differentiation control. Proc. Natl. Acad. Sci. Unit. States Am..

[10] O'Connell R.M., Chaudhuri A.A., Rao D.S., Gibson W.S., Balazs A.B., Baltimore D. (2010). MicroRNAs enriched in hematopoietic stem cells differentially regulate long-term hematopoietic output. Proc. Natl. Acad. Sci. U. S. A..

[11] Petriv O.I., Kuchenbauer F., Delaney A.D., Lecault V., White A., Kent D. (2010). Comprehensive microRNA expression profiling of the hematopoietic hierarchy. Proc. Natl. Acad. Sci. U. S. A..

[12] Chen C.-Z., Li L., Lodish H.F., Bartel D.P. (2004). MicroRNAs modulate hematopoietic lineage differentiation. science.

[13] Kurkewich J.L., Boucher A., Klopfenstein N., Baskar R., Kapur R., Dahl R. (2018). The mirn23a and mirn23b microrna clusters are necessary for proper hematopoietic progenitor cell production and differentiation. Exp. Hematol..

[14] Han Y.C., Park C.Y., Bhagat G., Zhang J., Wang Y., Fan J.B. (2010). microRNA-29a induces aberrant self-renewal capacity in hematopoietic progenitors, biased myeloid development, and acute myeloid leukemia. J. Exp. Med..

[15] Felli N., Fontana L., Pelosi E., Botta R., Bonci D., Facchiano F. (2005). MicroRNAs 221 and 222 inhibit normal erythropoiesis and erythroleukemic cell growth via kit receptor down-modulation. Proc. Natl. Acad. Sci. Unit. States Am..

[16] Garzon R., Pichiorri F., Palumbo T., Iuliano R., Cimmino A., Aqeilan R. (2006). MicroRNA fingerprints during human megakaryocytopoiesis. Proc. Natl. Acad. Sci. Unit. States Am..

[17] Fazi F., Rosa A., Fatica A., Gelmetti V., De Marchis M.L., Nervi C. (2005). A minicircuitry comprised of microRNA-223 and transcription factors NFI-A and C/EBPα regulates human granulopoiesis. Cell.

[18] Zhou B., Wang S., Mayr C., Bartel D.P., Lodish H.F. (2007). miR-150, a microRNA expressed in mature B and T cells, blocks early B cell development when expressed prematurely. Proc. Natl. Acad. Sci. Unit. States Am..

[19] Xiao C., Calado D.P., Galler G., Thai T.-H., Patterson H.C., Wang J. (2007). MiR-150 controls B cell differentiation by targeting the transcription factor c-Myb. Cell.

[20] Guo S., Lu J., Schlanger R., Zhang H., Wang J.Y., Fox M.C. (2010). MicroRNA miR-125a controls hematopoietic stem cell number. Proc. Natl. Acad. Sci. U. S. A..

[21] Ghafouri-Fard S., Niazi V., Taheri M. (2020). Role of miRNAs in conveying message of stem cells via extracellular vesicles. Exp. Mol. Pathol..

[22] Salvucci O., Jiang K., Gasperini P., Maric D., Zhu J., Sakakibara S. (2012). MicroRNA126 contributes to granulocyte colony-stimulating factor-induced hematopoietic progenitor cell mobilization by reducing the expression of vascular cell adhesion molecule 1. Haematologica.

[23] Niazi V., Parseh B., Ahani M., Karami F., Gilanchi S., Atarodi K. (2020). Communication between stromal and hematopoietic stem cell by exosomes in normal and malignant bone marrow niche. Biomed. Pharmacother..

[24] Guo S., Lu J., Schlanger R., Zhang H., Wang J.Y., Fox M.C. (2010). MicroRNA miR-125a controls hematopoietic stem cell number. Proc. Natl. Acad. Sci. Unit. States Am..

[25] Han Y.-C., Park C.Y., Bhagat G., Zhang J., Wang Y., Fan J.-B. (2010). microRNA-29a induces aberrant self-renewal capacity in hematopoietic progenitors, biased myeloid development, and acute myeloid leukemia. J. Exp. Med..

[26] Chen J.-F., Mandel E.M., Thomson J.M., Wu Q., Callis T.E., Hammond S.M. (2006). The role of microRNA-1 and microRNA-133 in skeletal muscle proliferation and differentiation. Nat. Genet..

[27] O'Connell R.M., Chaudhuri A.A., Rao D.S., Gibson W.S., Balazs A.B., Baltimore D. (2010). MicroRNAs enriched in hematopoietic stem cells differentially regulate long-term hematopoietic output. Proc. Natl. Acad. Sci. Unit. States Am..

[28] Bissels U., Wild S., Tomiuk S., Hafner M., Scheel H., Mihailovic A. (2011). Combined characterization of microRNA and mRNA profiles delineates early differentiation pathways of CD133+ and CD34+ hematopoietic stem and progenitor cells. Stem Cell..

[29] Rao D.S., O'Connell R.M., Chaudhuri A.A., Garcia-Flores Y., Geiger T.L., Baltimore D. (2010). MicroRNA-34a perturbs B lymphocyte development by repressing the forkhead box transcription factor Foxp1. Immunity.

[30] Tenedini E., Roncaglia E., Ferrari F., Orlandi C., Bianchi E., Bicciato S. (2010). Integrated analysis of microRNA and mRNA expression profiles in physiological myelopoiesis: role of hsa-mir-299-5p in CD34+ progenitor cells commitment. Cell Death Dis..

[31] Kurkewich J.L., Boucher A., Klopfenstein N., Baskar R., Kapur R., Dahl R. (2018). The mirn23a and mirn23b microrna clusters are necessary for proper hematopoietic progenitor cell production and differentiation. Exp. Hematol..

[32] Zhang L., Sankaran V., Lodish H. (2012). MicroRNAs in erythroid and megakaryocytic differentiation and megakaryocyte–erythroid progenitor lineage commitment. Leukemia.

[33] Hu H., Li Y., Gu J., Zhu X., Dong D., Yao J. (2010). Antisense oligonucleotide against miR-21 inhibits migration and induces apoptosis in leukemic K562 cells. Leuk. Lymphoma.

[34] Jiang X., Hu C., Arnovitz S., Bugno J., Yu M., Zuo Z. (2016). miR-22 has a potent anti-tumour role with therapeutic potential in acute myeloid leukaemia. Nat. Commun..

[35] Starczynowski D.T., Kuchenbauer F., Argiropoulos B., Sung S., Morin R., Muranyi A. (2010). Identification of miR-145 and miR-146a as mediators of the 5q–syndrome phenotype. Nat. Med..

[36] Girardot M., Pecquet C., Boukour S., Knoops L., Ferrant A., Vainchenker W. (2010). miR-28 is a thrombopoietin receptor targeting microRNA detected in a fraction of myeloproliferative neoplasm patient platelets. Blood, The Journal of the American Society of Hematology.

[37] Ben-Ami O., Pencovich N., Lotem J., Levanon D., Groner Y. (2009). A regulatory interplay between miR-27a and Runx1 during megakaryopoiesis. Proc. Natl. Acad. Sci. Unit. States Am..

[38] Dore L.C., Amigo J.D., Dos Santos C.O., Zhang Z., Gai X., Tobias J.W. (2008). A GATA-1-regulated microRNA locus essential for erythropoiesis. Proc. Natl. Acad. Sci. Unit. States Am..

[39] Rasmussen K.D., Simmini S., Abreu-Goodger C., Bartonicek N., Di Giacomo M., Bilbao-Cortes D. (2010). The miR-144/451 locus is required for erythroid homeostasis. J. Exp. Med..

[40] Patrick D.M., Zhang C.C., Tao Y., Yao H., Qi X., Schwartz R.J. (2010). Defective erythroid differentiation in miR-451 mutant mice mediated by 14-3-3ζ. Genes Dev..

[41] Luo M., Jeong M., Sun D., Park H.J., Rodriguez B.A., Xia Z. (2015). Long non-coding RNAs control hematopoietic stem cell function. Cell Stem Cell.

[42] Ebralidze A.K., Guibal F.C., Steidl U., Zhang P., Lee S., Bartholdy B. (2008). 1 expression is modulated by the balance of functional sense and antisense RNAs regulated by a shared cis-regulatory element. Genes Dev..

[43] Dahl R., Walsh J.C., Lancki D., Laslo P., Iyer S.R., Singh H. (2003). Regulation of macrophage and neutrophil cell fates by the PU. 1: C/EBPα ratio and granulocyte colony-stimulating factor. Nat. Immunol..

[44] Paralkar V.R., Mishra T., Luan J., Yao Y., Kossenkov A.V., Anderson S.M. (2014). Lineage and species-specific long noncoding RNAs during erythro-megakaryocytic development. Blood. The Journal of the American Society of Hematology.

[45] Wagner L.A., Christensen C.J., Dunn D.M., Spangrude G.J., Georgelas A., Kelley L. (2007). EGO, a novel, noncoding RNA gene, regulates eosinophil granule protein transcript expression. Blood.

[46] Zhang X., Lian Z., Padden C., Gerstein M.B., Rozowsky J., Snyder M. (2009). A myelopoiesis-associated regulatory intergenic noncoding RNA transcript within the human HOXA cluster. Blood. The Journal of the American Society of Hematology.

[47] Villamizar O., Chambers C.B., Mo Y.-Y., Torry D.S., Hofstrand R., Riberdy J.M. (2016). Fas-antisense long noncoding RNA is differentially expressed during maturation of human erythrocytes and confers resistance to Fas-mediated cell death. Blood Cell Mol. Dis..

[48] Hu W., Yuan B., Flygare J., Lodish H.F. (2011). Long noncoding RNA-mediated anti-apoptotic activity in murine erythroid terminal differentiation. Genes Dev..

[49] Ranzani V., Rossetti G., Panzeri I., Arrigoni A., Bonnal R.J., Curti S. (2015). The long intergenic noncoding RNA landscape of human lymphocytes highlights the regulation of T cell differentiation by linc-MAF-4. Nat. Immunol..

[50] Zhou J., Xu J., Zhang L., Liu S., Ma Y., Wen X. (2019). Combined single-cell profiling of lncRNAs and functional screening reveals that H19 is pivotal for embryonic hematopoietic stem cell development. Cell Stem Cell.

[51] Yildirim E., Kirby J.E., Brown D.E., Mercier F.E., Sadreyev R.I., Scadden D.T. (2013). Xist RNA is a potent suppressor of hematologic cancer in mice. Cell.

[52] Qian P., He X.C., Paulson A., Li Z., Tao F., Perry J.M. (2016). The Dlk1-Gtl2 locus preserves LT-HSC function by inhibiting the PI3K-mTOR pathway to restrict mitochondrial metabolism. Cell Stem Cell.

[53] Alvarez-Dominguez J.R., Hu W., Yuan B., Shi J., Park S.S., Gromatzky A.A. (2014). Global discovery of erythroid long noncoding RNAs reveals novel regulators of red cell maturation. Blood, The Journal of the American Society of Hematology.

[54] Zhang X., Weissman S.M., Newburger P.E. (2014). Long intergenic non-coding RNA HOTAIRM1 regulates cell cycle progression during myeloid maturation in NB4 human promyelocytic leukemia cells. RNA Biol..

[55] Díaz-Beyá M., Brunet S., Nomdedéu J., Pratcorona M., Cordeiro A., Gallardo D. (2015). The lincRNA HOTAIRM1, located in the HOXA genomic region, is expressed in acute myeloid leukemia, impacts prognosis in patients in the intermediate-risk cytogenetic category, and is associated with a distinctive microRNA signature. Oncotarget.

[56] Wei S., Zhao M., Wang X., Li Y., Wang K. (2016). PU. 1 controls the expression of long noncoding RNA HOTAIRM1 during granulocytic differentiation. J. Hematol. Oncol..

[57] Chen Z.-H., Wang W.-T., Huang W., Fang K., Sun Y.-M., Liu S.-R. (2017). The lncRNA HOTAIRM1 regulates the degradation of PML-RARA oncoprotein and myeloid cell differentiation by enhancing the autophagy pathway. Cell Death Differ..

[58] Vickers N.J. (2017). Animal communication: when i'm calling you, will you answer too?. Curr. Biol..

[59] Linder P., Jankowsky E. (2011). From unwinding to clamping—the DEAD box RNA helicase family. Nat. Rev. Mol. Cell Biol..

[60] Huang W., Thomas B., Flynn R.A., Gavzy S.J., Wu L., Kim S.V. (2018). Erratum to: DDX5 and its associated lncRNA Rmrp modulate TH17 cell effector functions (Nature,(2015), 528, 7583,(517-522), 10.1038/nature16193). Nature.

[61] Zan H., Casali P. (2015). Epigenetics of peripheral B-cell differentiation and the antibody response. Front. Immunol..

[62] Gomez J.A., Wapinski O.L., Yang Y.W., Bureau J.-F., Gopinath S., Monack D.M. (2013). The NeST long ncRNA controls microbial susceptibility and epigenetic activation of the interferon-γ locus. Cell.

[63] Peng X., Gralinski L., Armour C.D., Ferris M.T., Thomas M.J., Proll S. (2010). Unique signatures of long noncoding RNA expression in response to virus infection and altered innate immune signaling. mBio.

[64] Yagi R., Zhu J., Paul W.E. (2011). An updated view on transcription factor GATA3-mediated regulation of Th1 and Th2 cell differentiation. Int. Immunol..

[65] Hu G., Tang Q., Sharma S., Yu F., Escobar T.M., Muljo S.A. (2013). Expression and regulation of intergenic long noncoding RNAs during T cell development and differentiation. Nat. Immunol..

[66] Zhang H., Nestor C.E., Zhao S., Lentini A., Bohle B., Benson M. (2013). Profiling of human CD4+ T-cell subsets identifies the TH2-specific noncoding RNA GATA3-AS1. J. Allergy Clin. Immunol..

[67] Spurlock C.F., Tossberg J.T., Guo Y., Collier S.P., Crooke P.S., Aune T.M. (2015). Expression and functions of long noncoding RNAs during human T helper cell differentiation. Nat. Commun..

[68] Schlaphoff V., Lunemann S., Suneetha P.V., Jaroszewicz J., Grabowski J., Dietz J. (2011). Dual function of the NK cell receptor 2B4 (CD244) in the regulation of HCV-specific CD8+ T cells. PLoS Pathog..

[69] Wang Y., Zhong H., Xie X., Chen C.Y., Huang D., Shen L. (2015). Long noncoding RNA derived from CD244 signaling epigenetically controls CD8+ T-cell immune responses in tuberculosis infection. Proc. Natl. Acad. Sci. Unit. States Am..

[70] Sharma S., Findlay G.M., Bandukwala H.S., Oberdoerffer S., Baust B., Li Z. (2011). Dephosphorylation of the nuclear factor of activated T cells (NFAT) transcription factor is regulated by an RNA-protein scaffold complex. Proc. Natl. Acad. Sci. Unit. States Am..

[71] Eis P.S., Tam W., Sun L., Chadburn A., Li Z., Gomez M.F. (2005). Accumulation of miR-155 and BIC RNA in human B cell lymphomas. Proc. Natl. Acad. Sci. Unit. States Am..

[72] Tam W. (2001). Identification and characterization of human BIC, a gene on chromosome 21 that encodes a noncoding RNA. Gene.

[73] Elton T.S., Selemon H., Elton S.M., Parinandi N.L. (2013). Regulation of the MIR155 host gene in physiological and pathological processes. Gene.

[74] Calin G.A., Liu C-g, Ferracin M., Hyslop T., Spizzo R., Sevignani C. (2007). Ultraconserved regions encoding ncRNAs are altered in human leukemias and carcinomas. Canc. Cell.

[75] Swalwell H., Latimer J., Haywood R.M., Birch-Machin M.A. (2012). Investigating the role of melanin in UVA/UVB-and hydrogen peroxide-induced cellular and mitochondrial ROS production and mitochondrial DNA damage in human melanoma cells. Free Radic. Biol. Med..

[76] Jiang R., Tang J., Chen Y., Deng L., Ji J., Xie Y. (2017). The long noncoding RNA lnc-EGFR stimulates T-regulatory cells differentiation thus promoting hepatocellular carcinoma immune evasion. Nat. Commun..

[77] Vigneau S., Rohrlich P.-S., Brahic M., Bureau J.-F. (2003). Tmevpg1, a candidate gene for the control of Theiler's virus persistence, could be implicated in the regulation of gamma interferon. J. Virol..

[78] Winkle M., Kluiver J., Diepstra A., van den Berg A. (2017). Emerging roles for long noncoding RNAs in B-cell development and malignancy. Crit. Rev. Oncol.-Hematol..

[79] Haasch D., Chen Y.-W., Reilly R.M., Chiou X.G., Koterski S., Smith M.L. (2002). T cell activation induces a noncoding RNA transcript sensitive to inhibition by immunosuppressant drugs and encoded by the proto-oncogene. BIC. Cellular immunology.

[80] Collier S.P., Collins P.L., Williams C.L., Boothby M.R., Aune T.M. (2012). Cutting edge: influence of Tmevpg1, a long intergenic noncoding RNA, on the expression of Ifng by Th1 cells. J. Immunol..

[81] Carpenter S., Aiello D., Atianand M.K., Ricci E.P., Gandhi P., Hall L.L. (2013). A long noncoding RNA mediates both activation and repression of immune response genes. science.

[82] Aoki K., Harashima A., Sano M., Yokoi T., Nakamura S., Kibata M. (2010). A thymus-specific noncoding RNA, Thy-ncR1, is a cytoplasmic riboregulator of MFAP4 mRNA in immature T-cell lines. BMC Mol. Biol..

[83] Venkatraman A., He X.C., Thorvaldsen J.L., Sugimura R., Perry J.M., Tao F. (2013). Maternal imprinting at the H19–Igf2 locus maintains adult haematopoietic stem cell quiescence. Nature.

[84] Stadtfeld M., Apostolou E., Akutsu H., Fukuda A., Follett P., Natesan S. (2010). Aberrant silencing of imprinted genes on chromosome 12qF1 in mouse induced pluripotent stem cells. Nature.

[85] Luo S., Lu J.Y., Liu L., Yin Y., Chen C., Han X. (2016). Divergent lncRNAs regulate gene expression and lineage differentiation in pluripotent cells. Cell stem cell.

[86] Zhou J., Xu J., Zhang L., Liu S., Ma Y., Wen X. (2019). Combined single-cell profiling of lncRNAs and functional screening reveals that H19 is pivotal for embryonic hematopoietic stem cell development. Cell Stem Cell.

[87] McHugh C.A., Chen C.-K., Chow A., Surka C.F., Tran C., McDonel P. (2015). The Xist lncRNA interacts directly with SHARP to silence transcription through HDAC3. Nature.

[88] Wang P., Xue Y., Han Y., Lin L., Wu C., Xu S. (2014). The STAT3-binding long noncoding RNA lnc-DC controls human dendritic cell differentiation. Science.

[89] Rapicavoli N.A., Qu K., Zhang J., Mikhail M., Laberge R.-M., Chang H.Y. (2013). A mammalian pseudogene lncRNA at the interface of inflammation and anti-inflammatory therapeutics. elife.

[90] Li Z., Chao T.-C., Chang K.-Y., Lin N., Patil V.S., Shimizu C. (2014). The long noncoding RNA THRIL regulates TNFα expression through its interaction with hnRNPL. Proc. Natl. Acad. Sci. Unit. States Am..

[91] Krawczyk M., Emerson B.M. (2014). p50-associated COX-2 extragenic RNA (PACER) activates COX-2 gene expression by occluding repressive NF-κB complexes. elife.

[92] Liu B., Sun L., Liu Q., Gong C., Yao Y., Lv X. (2015). A cytoplasmic NF-κB interacting long noncoding RNA blocks IκB phosphorylation and suppresses breast cancer metastasis. Canc. Cell.

[93] Sun S., Del Rosario B.C., Szanto A., Ogawa Y., Jeon Y., Lee J.T. (2013). Jpx RNA activates Xist by evicting CTCF. Cell.

[94] Arriaga-Canon C., Fonseca-Guzmán Y., Valdes-Quezada C., Arzate-Mejía R., Guerrero G., Recillas-Targa F. (2014). A long non-coding RNA promotes full activation of adult gene expression in the chicken α-globin domain. Epigenetics.

[95] Kino T., Hurt D.E., Ichijo T., Nader N., Chrousos G.P. (2010). Noncoding RNA gas5 is a growth arrest–and starvation-associated repressor of the glucocorticoid receptor. Sci. Signal..

[96] Ørom U.A., Derrien T., Beringer M., Gumireddy K., Gardini A., Bussotti G. (2010). Long noncoding RNAs with enhancer-like function in human cells. Cell.

[97] Benetatos L., Hatzimichael E., Dasoula A., Dranitsaris G., Tsiara S., Syrrou M. (2010). CpG methylation analysis of the MEG3 and SNRPN imprinted genes in acute myeloid leukemia and myelodysplastic syndromes. Leuk. Res..

[98] Willingham A., Orth A., Batalov S., Peters E., Wen B., Aza-Blanc P. (2005). A strategy for probing the function of noncoding RNAs finds a repressor of NFAT. Science.

[99] Sehgal L., Mathur R., Braun F.K., Wise J.F., Berkova Z., Neelapu S. (2014). FAS-antisense 1 lncRNA and production of soluble versus membrane Fas in B-cell lymphoma. Leukemia.

[100] Monticelli S., Ansel K.M., Xiao C., Socci N.D., Krichevsky A.M., Thai T.-H. (2005). MicroRNA profiling of the murine hematopoietic system. Genome Biol..

